# Modified Lipoproteins Induce Arterial Wall Inflammation During Atherogenesis

**DOI:** 10.3389/fcvm.2022.841545

**Published:** 2022-03-03

**Authors:** Martina B. Lorey, Katariina Öörni, Petri T. Kovanen

**Affiliations:** ^1^Atherosclerosis Research Laboratory, Wihuri Research Institute, Helsinki, Finland; ^2^Molecular and Integrative Biosciences, Faculty of Biological and Environmental Sciences, University of Helsinki, Helsinki, Finland

**Keywords:** modified lipoproteins, inflammation, atherogenesis, endothelial dysfunction, foam cell

## Abstract

Circulating apolipoprotein B-containing lipoproteins, notably the low-density lipoproteins, enter the inner layer of the arterial wall, the intima, where a fraction of them is retained and modified by proteases, lipases, and oxidizing agents and enzymes. The modified lipoproteins and various modification products, such as fatty acids, ceramides, lysophospholipids, and oxidized lipids induce inflammatory reactions in the macrophages and the covering endothelial cells, initiating an increased leukocyte diapedesis. Lipolysis of the lipoproteins also induces the formation of cholesterol crystals with strong proinflammatory properties. Modified and aggregated lipoproteins, cholesterol crystals, and lipoproteins isolated from human atherosclerotic lesions, all can activate macrophages and thereby induce the secretion of proinflammatory cytokines, chemokines, and enzymes. The extent of lipoprotein retention, modification, and aggregation have been shown to depend largely on differences in the composition of the circulating lipoprotein particles. These properties can be modified by pharmacological means, and thereby provide opportunities for clinical interventions regarding the prevention and treatment of atherosclerotic vascular diseases.

## Introduction

Atherosclerosis develops in the inner layer of the arterial wall, the intima. Atherosclerosis-prone locations, such as bifurcations of the coronary arteries, are characterized by a thick extracellular matrix containing collagen, elastin, and proteoglycans, and the presence of increased numbers of lipid-laden macrophages since childhood (1–3). Apolipoprotein B (apoB)-containing lipoproteins, i.e., LDL and remnant lipoproteins, such as small VLDL, IDL, and chylomicron remnants, cross the endothelial cell layer from the circulation into the intima (4), where they can become entrapped by the dense extracellular matrix (5–7).

A fraction of the lipoproteins can be modified in the intima by oxidizing agents and enzymes, proteases, and lipases secreted by local cells (5, 6). Such modifications of the lipoprotein structure have been shown to induce aggregation and fusion of lipoprotein particles (8, 9). Atherosclerotic lesions contain lipoproteins that show signs of extensive oxidation, proteolysis, and lipolysis of the various lipid molecules and that are often aggregated [recently reviewed in (10)]. The modified lipoproteins can be taken up by macrophages, which are converted into foam cells. The initial lesions of atherosclerosis, the fatty streaks, are characterized by the presence of numerous foam cells and can appear at any age, independent of risk factors for atherosclerotic cardiovascular disease (11). Moreover, in contrast to more advanced lesions, the fatty streaks are potentially fully reversible (12).

During atherogenesis, macrophages, dendritic cells, mast cells, and T lymphocytes are recruited into the intima in increasing numbers (13, 14). In the developing lesions, the macrophage foam cells die whereby the remains of the dead cells and the lipids they contained form a “necrotic lipid core.” As the necrotic core of the atherosclerotic plaque grows, the lumen of the coronary artery progressively narrows, and the myocardial tissue becomes progressively hypoxic (15). Simultaneously, the intimal layer between the endothelium and the necrotic lipid core, called the fibrous or collagenous cap, becomes progressively thinner (16) due to a decrease in the numbers of extracellular matrix producing smooth muscle cells (SMCs) and due to enhanced secretion of collagen-degrading enzymes by macrophages and macrophage foam cells. A thin-cap atherosclerotic lesion is vulnerable to rupture and it often causes an acute occluding thrombus in the coronary artery with subsequent ischemic myocardial damage, i.e., an acute myocardial infarction (17).

Modification of lipoproteins in the intima is one of the first steps in atherogenesis. The modified lipoproteins and the various proinflammatory molecules generated during lipoprotein modification influence the local cells and can initiate a vicious cycle of lipoprotein modification, lipid accumulation, and inflammation [reviewed in (18)]. In this review we discuss the distinct types of lipoprotein modifications and the consequences of the lipoprotein modifications on local cells as well as the development and progression of atherosclerosis.

### Lipoprotein Modification

**Figure 1:**
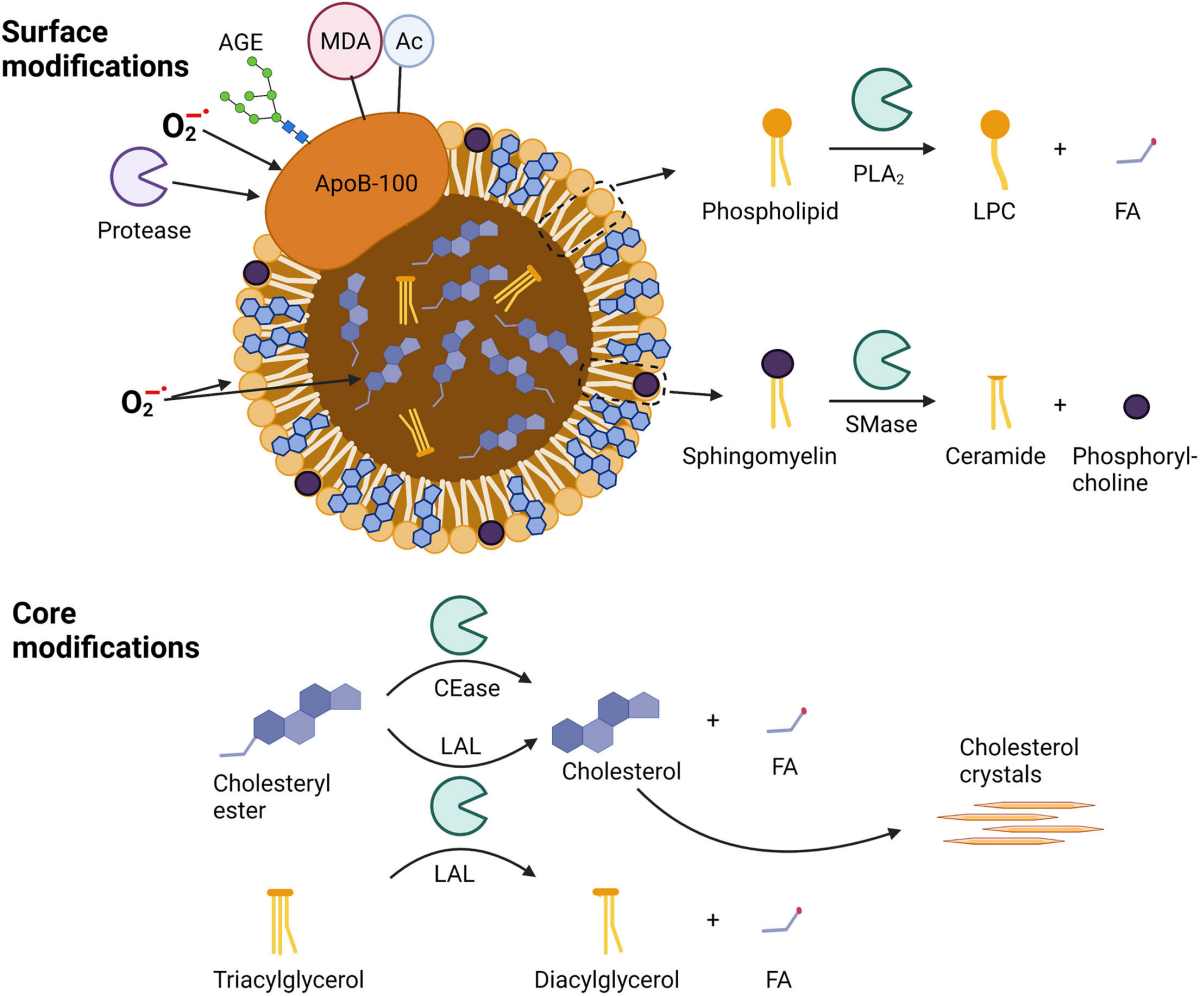
Sites of modification in apoB-100-containing lipoproteins. Surface modifications include modifications of apo-B100 by proteases, oxidation by superoxide anion radicals (${\text{O}}_{2}^{-}$), glycosylation by advanced glycosylation end-products (AGEs), binding of malondialdehyde (MDA) adducts, or acetylation (Ac). Phospholipids can be oxidized as well as hydrolyzed by phospholipase A2 (PLA_2_) to lysophosphocholine (LPC) and a fatty acid (FA), while sphingomyelins can be hydrolyzed by sphingomyelinase (SMase) to yield ceramides and phosphorylcholines. Core modifications include cholesteryl ester oxidation and well as hydrolysis by cholesterol esterase (CEase) or lysosomal acid lipase (LAL) to yield unesterified cholesterol and a fatty acid (FA). Triacylglycerol can be hydrolyzed by LAL to diacylglycerol and a fatty acid (FA).

To cross the endothelium, apoB-containing lipoproteins can bind to activin receptor-like kinase 1 and scavenger receptor B1 on the surface of the endothelial cells (ECs) lining the vessels after which they are transported by transcytosis into the intima [reviewed in (19)]. In the intima, the lipoproteins bind via ionic interactions to the negatively charged proteoglycans of the extracellular matrix (5–7) and can be attacked by various proteases, lipases, and oxidizing agents and enzymes, as evidenced by lipoproteins isolated from atherosclerotic lesions having oxidized epitopes and showing signs of proteolysis and lipolysis [reviewed in (8, 10)]. Of note, the oxidatively modified particles from human atherosclerotic lesions are more extensively modified than in the corresponding plasma, raising the possibility that the changes have been initiated already in the plasma (20). Also, each of the above-listed types of modification has been shown to induce lipoprotein aggregation *in vitro* (8). Aggregation and fusion of lipoprotein particles increase their affinities of their affinities to proteoglycans its and induce foam cell formation (8). Moreover, the aggregation-susceptibility of LDL particles has been linked with future cardiovascular events in patients having established atherosclerosis (21, 22). The relative importance of the various types of lipoprotein modification in atherogenesis remains to be determined, but regarding the inflammatory potential of the modified lipoproteins, the products generated during lipoprotein modification (Figure 1) may be particularly relevant, as will be discussed in this review.

Atherosclerotic lesions contain both neutral and acidic proteases and lipases that can modify lipoprotein particles. Among the proteases are serine proteases, cathepsins, and metalloproteinases that have been shown to degrade apoB-100 (23–26). Of note, these proteases may also degrade the structural proteins like collagens in the extracellular matrix and may thus lead to cap-thinning of the plaques [reviewed in (27)]. The extent of proteolytic degradation of the protein components of the lipoproteins depends on the protease: plasmin, for example, can induce fragmentation of apoB-100 of LDL and does not induce LDL aggregation and fusion, while cathepsins proteolyze apoB-100 of LDL into small fragments and induce the generation of large, fused particles (24, 28, 29). Proteolysis can also promote lipolytic modifications of the lipoproteins (30). An example of the combination of proteolysis and lipolysis is enzymatically modified LDL (E-LDL), which is generated by treating LDL with a protease, such as matrix metalloproteinase 9 or cathepsin H, and cholesteryl esterase (31–33).

Extensive oxidation can result in the fragmentation of apoB (34). Oxidation also leads to changes in the lipoprotein lipids, leads to the generation of malondialdehyde adducts in the lysine residues of the proteins, and induces the generation of oxidation-specific epitopes on the lipoproteins. Unlike proteolytic and lipolytic modifications, oxidation decreases the interaction of lipoproteins with proteoglycans. However, oxidized LDL (oxLDL) has been shown to bind to decorin-coated collagen via lipoprotein lipase thereby leading to extracellular accumulation of oxLDL (35). OxLDL also induces foam cell formation via interaction with various scavenger receptors on macrophages (36, 37).

Phospholipase A_2_ (PLA_2_) is an enzyme that hydrolyzes phospholipids generating non-esterified fatty acids (NEFAs) and lysophospholipids. PLA_2_ comprises several subtypes and can be found extracellularly as secreted PLA_2_s, intracellularly as cytosolic PLA_2_s, and in the bloodstream as lipoprotein-associated PLA_2_ (Lp-PLA_2_) (38). Lp-PLA_2_ is produced by hepatocytes, and also by inflammatory cells such as macrophages, foam cells, and mast cells, as well as T-lymphocytes in atherosclerotic plaques, and in the circulation it is mainly associated with LDL (39, 40). The various PLA_2_s have distinct specificities, group IIa, group V, and group X PLA_2_s being able to avidly hydrolyze phosphatidylcholine on LDL particles, while Lp-PLA_2_ preferably hydrolyzes oxidized phospholipids. Lp-PLA_2_ and several types of secretory PLA_2_s have been found in atherosclerotic plaques and in the normal arterial intima (38, 41–43). Modification of LDL, IDL, and VLDL with PLA_2_ increases their affinities to proteoglycans *in vitro* (44, 45), and thereby potentially enhances the retention of LDL particles in the vessel wall. PLA_2_-modified LDL also promotes macrophage foam cell formation (46, 47).

Treatment of LDL with an enzyme secreted by different cell types within the arterial intima, namely the sphingomyelinase (SMase), leads to conformational changes in apoB-100 and induces lipoprotein particle aggregation with ensuing increased proteoglycan binding (44, 48, 49). Secretory SMase implicated in atherogenesis has an acidic pH optimum, but can hydrolyze oxidized, PLA_2_-treated, or proteolyzed LDL at neutral pH (30, 49). In addition, VLDL particles appear to be very susceptible to hydrolysis and aggregation by SMase (50, 51). Interestingly, ceramide, the lipolytic product of SMase action, is found exclusively in very large aggregates (49), such as those formed *in vitro* by modification of LDL by SMase at pH 5.5-6 (48). Generally, the sizes of LDL aggregates after *in vitro* modification differ substantially depending on the type of modification and the conditions used (48, 52). Although the aggregate size can affect aggregate uptake by, e.g., leukocytes (53), no studies have been performed to systematically study the effect of aggregate size on their pro-inflammatory properties either *in vitro* or *in vivo*.

Hydrolysis of the core lipids (cholesteryl esters and triglycerides) of lipoproteins has the potential to induce the generation of large amounts of NEFAs. The lipolytic hydrolysis is enhanced if the surface of the particles is also modified, e.g., by proteolysis, which allows penetration of some of the core lipids into the surface membrane of the lipoproteins (54). The E-LDL is an example of such multiply modified lipoprotein and antibodies generated against E-LDL bind to extracellular lipids in atherosclerotic lesions (55). Hydrolysis of cholesteryl esters has the potential to generate large amounts of cholesterol and lead to the generation of cholesterol crystals (56), which are a typical feature of very advanced atherosclerotic lesions (14). More recently, small cholesterol crystals have been identified also in early atherosclerotic lesions (57).

### Complement

The complement system is a tightly regulated proteolytic cascade, a key component of innate immunity comprising over 30 soluble and membrane-bound proteins that responds rapidly to clear invading pathogens and damaged host cells, as well as to limit tissue destruction and initiate tissue healing. Complement activation is triggered by exposure to endogenous danger molecules that are released from damaged or dying cells and called danger-associated molecular patterns (DAMPs) (58). Depending on the initiation, the complement cascade follows either the lectin, classical, or alternative pathway. While these pathways have different activation cascades, they all converge downstream with the formation of their respective Complement (C) 3 convertases and the generation of C3a and C3b. If the complement activation is of sufficient magnitude, the membrane attack complex C5b-9 is formed. This important innate immune effector has pore-forming, lytic properties designed to destroy pathogens and damaged cells (59–61).

Circulating C3 levels were found to be significantly elevated in patients having familial hypercholesterolemia with subclinical coronary atherosclerosis, however, there was no correlation between circulating C3 levels and increased plaque burden, indicating a local regulation of the C3 in atherosclerotic arteries (62). Indeed, active components of the C3 complement are found within the extracellular matrix of human arteries (63). Complement components, including C3 were also identified in extracellular lipoproteins isolated from human carotid atherosclerotic lesions (56), and E-LDL was shown to colocalize with C5b-9 in human atherosclerotic lesions (55). Potential triggers of complement activation in the arterial wall include modified lipoproteins, cholesterol crystals, antigen-antibody immune complexes, C-reactive protein (CRP), and apoptotic cells [reviewed in (59, 63–65)]. Importantly, aggregated and fused lipoproteins isolated from human atherosclerotic lesions possess complement-activating properties (66). These particles do not contain immunoreactive apoB-100 but contain both esterified and unesterified cholesterol, and have sizes ranging from 100 to 500 nm (33). E-LDL with or without bound CRP, oxLDL, malondialdehyde-LDL, and oxLDL-immune complexes have been shown to induce complement activation [reviewed in (67)]. E-LDL, but not oxLDL, can trigger C1 activation and bind complement components (68, 69). Factor H, a major complement inhibitor, colocalizes in human atherosclerotic lesions with C3d in the subendothelial proteoglycan-rich layer, while C5b-9 is found in deeper areas of atherosclerotic lesions suggesting that in different areas of the lesions the components may differ in their abilities to regulate complement activation (63).

### Foam Cell Formation

Foam cells are the histological hallmark of atherosclerotic lesions from the very beginning of the lesion development, and their contribution to atherogenesis remains throughout the lesion progression (13, 15). They are derived mostly from macrophages that take up modified and aggregated lipoproteins and lipoprotein-immune complexes via several different mechanisms (36). In addition to the macrophages, also vascular SMCs take up modified lipoproteins and can become foam cells (70, 71). The various uptake mechanisms include scavenger receptors that are important particularly in the uptake of oxLDL, Fc-gamma receptors interacting with immune complexes, cell surface proteoglycan-aided uptake of PLA_2_-modified LDL, and phagocytosis of aggregated LDL and even cholesterol crystals (46, 72–75). Native VLDL remnant particles have been shown to induce foam cell formation through the VLDL receptor pathway in macrophages, and modification of VLDL particles by acetylation leads to macrophage foam cell formation via scavenger receptor-mediated uptake of the modified particles (76).

A specific uptake mechanism in macrophages has been reported for large LDL aggregates, such as SMase-modified LDL particles. Thus, aggregated LDL triggers the formation of extracellular surface-connected compartments into which the macrophages (77) and dendritic cells (78) secrete lysosomal enzymes, such as lysosomal acid lipase (LAL) (78–80). The process leads to a partial hydrolysis of the aggregated LDL outside the cells and to internalization of the partially hydrolyzed lipoproteins into lysosomes, where they are fully hydrolyzed. Actin polymerization and Toll-like receptor (TLR) 4 activation drive the intracellular catabolism of the aggregates (80, 81). In addition, treatment of LDL with macrophage-conditioned media containing catalytically active LAL induces LDL fusion and lipid droplet formation in VSMCs (54). LAL activity may be particularly prominent in the macrophage-rich acidic microenvironments observed in human atherosclerotic lesions (82).

**Figure 2:**
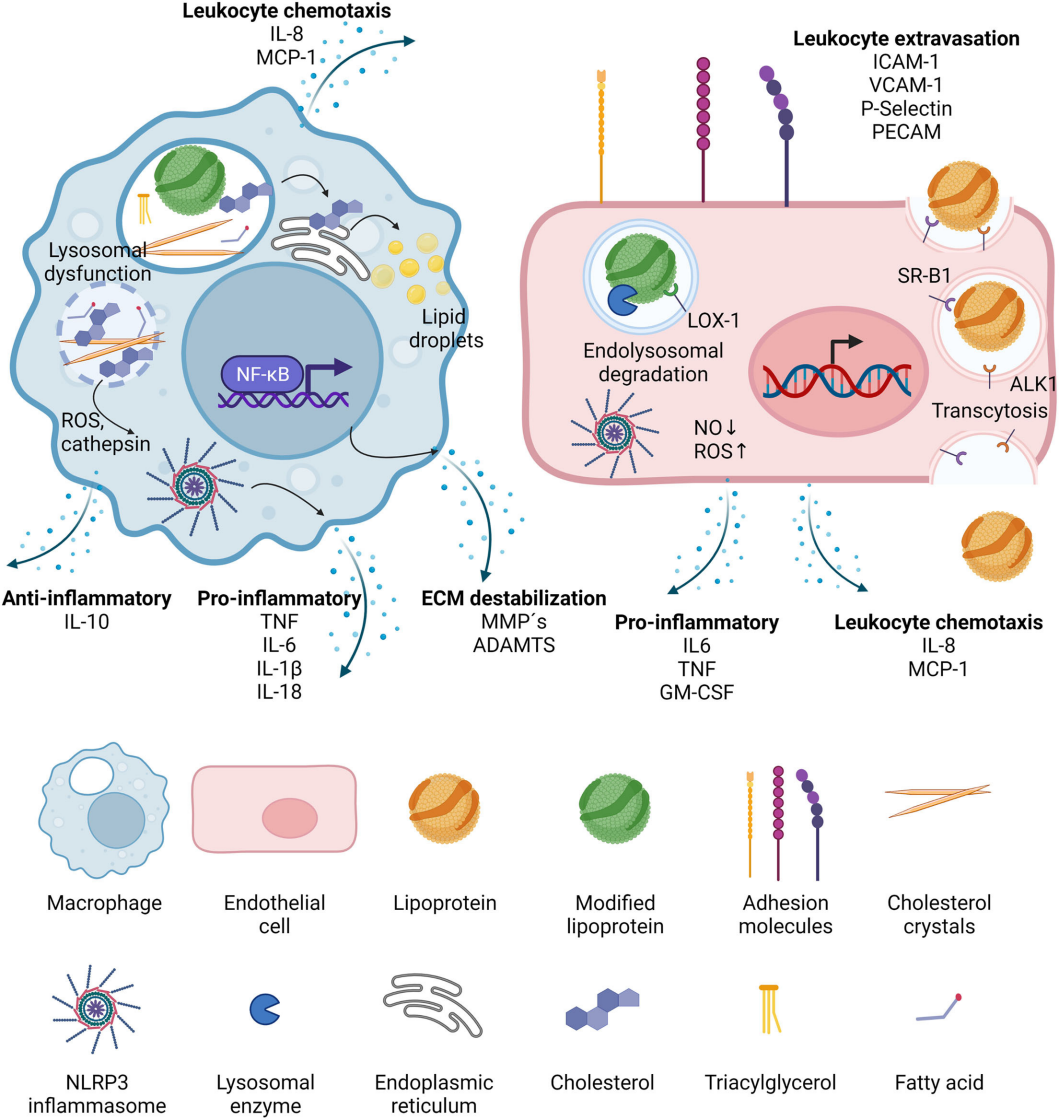
Effects of lipoproteins on macrophages and endothelial cells. Upon phagocytosis, modified lipoproteins are hydrolyzed in the lysosomes of macrophages. Cholesterol is then transported to the ER where it is packaged in lipid droplets for storage. It can also crystalize in the lysosomes, leading to lysosomal dysfunction and the release of reactive oxygen species (ROS) and cathepsins, which in turn activate cytosolic inflammasomes. NF-κB signaling is induced and the activated macrophages secrete leukocyte chemotactic molecules, pro- as well as anti-inflammatory cytokines, and proteases. In endothelial cells, lipoproteins can be transported through the cell via transcytosis, mediated by binding to scavenger receptor B1 (SR-B1) and activin-like kinase 1 (ALK1). If the lipoproteins have been modified, they can also bind to lectin-type oxidized LDL receptor 1 (LOX-1) and then targeted to the lysosomes after internalization. LOX-1 activation leads to decrease in nitric oxide (NO) and increase in reactive oxygen species (ROS), the latter being able to trigger NLRP3 inflammasome activation. Activated endothelial cells increase the expression of adhesion molecules which accelerates leukocyte extravasation, and they also secrete leukocyte chemotactic factors, as well as pro-inflammatory cytokines.

Once lipoproteins have reached the lysosomes, their components are completely hydrolyzed (Figure 2). Importantly, cholesteryl ester hydrolysis by LAL results in the formation of unesterified cholesterol, which will then be re-esterified in the cytoplasm and packaged into intracellular lipid droplets characteristic of foam cells (83). Uptake of aggregated LDL has been shown to lead to accumulation of ceroid, a product of lipid oxidation consisting of protein-lipid complexes, in lysosomes (84). Lysosomal oxidation is likely mediated by iron and can be inhibited by cysteamine (85). On the other hand, uptake of oxLDL can result in lipid accumulation in the lysosomes (86, 87) and lead to the formation of cholesterol crystals in the lysosomes (88). However, the transformation of macrophages into foam cells may reduce their atheroinflammatory potential (89).

In vascular SMCs, foam cell formation due to uptake of native and modified VLDL was shown to depend on the activity of lipoprotein lipase (90), while the LDL receptor-related protein 1 plays a role in the uptake of aggregated LDL (91). Aggregated LDL particles increase the release of the soluble form of LDL receptor-related protein 1 from cultured vascular SMCs, and from human atherosclerotic plaques in an ex vivo model (92). Interestingly, the level of the soluble LDL receptor-related protein 1 in the circulating blood is increased in patients with coronary artery disease (92, 93). When compared with cultured human macrophages, cultured human SMCs express relatively low levels of LAL (94). Accordingly, in SMC foam cells, the ingested neutral lipids tend to accumulate within lysosomes, while in macrophages they appear as cytosolic droplets.

While complement activation is usually regarded to be pro-atherogenic, complement plays also a smoothing role in mediating the effects of foam cell formation. Thus, while the complement component C1q recognizes and opsonizes modified forms of LDL and promotes phagocytosis and efferocytosis in macrophage foam cells (95, 96), when bound to acLDL or oxLDL it reduces the release of proinflammatory cytokines from macrophage foam cells and atherosclerosis in mouse models (97). In addition, C1q downregulates both the levels and activities of apoptosis-related proteins in human and mouse macrophage foam cells, thereby leading to a measurable increase in the survival of these cells, which, again, could slow down the build-up of the necrotic core in the atherosclerotic plaques (95). Further, factor H, the main regulator of the alternative complement activation pathway, has been shown to bind to human monocytes and macrophage foam cells and has the potential to reduce complement activation and inflammation, and to increase the binding of apoE to these cells. It further increased cholesterol efflux, and cholesterol-loaded macrophages displayed reduced transcription of proinflammatory/proatherogenic factors and increased transcription of anti-inflammatory/anti-atherogenic factors, thereby indicating that apoE and factor H interact with monocytic cells in a concerted action and that this interaction tends to reduce complement activation and inflammation in atherosclerotic lesions (98).

## Modified Lipoproteins Induce Inflammation Via Innate Immune Cells

Continued accumulation of immunogenic modified lipoproteins and the attendant pro-inflammatory reactions in the arterial intima result in the development of atherosclerotic lesions (99). Thus, atherosclerosis is driven by a chronic, low-grade inflammation of the vascular wall that continuously attracts immune cells into the developing atherosclerotic plaques (100).

As the first step in atherogenesis, native lipoproteins enter the intima by transcytosis, get trapped and modified by enzymes secreted by resident macrophages and mast cells, as described above. Minimally modified lipoproteins from the circulation or modified lipoproteins from the intima can be internalized by ECs via the lectin-like oxidized LDL receptor 1 (LOX-1) and be destined for lysosomal degradation, which activates the ECs and initiates endothelial dysfunction [Figure 2, reviewed in (101)]. ECs also get activated by secreted cytokines and chemoattractants derived from activated intimal macrophages (101). The activated endothelium then secretes chemoattractants and increases the expression of adhesion molecules so aiding the tethering, rolling, and adhesion steps of the leukocyte diapedesis process. Within the intima, monocytes differentiate into macrophages or DCs, and the naïve T cells mature. Macrophages can be of pro-inflammatory (M1) or anti-inflammatory/pro-resolving (M2) phenotype, and their phenotype can change in response to alterations in their microenvironmental conditions [reviewed in (102)]. The above widely used dichotomous classification of macrophages into pro- and anti-inflammatory phenotypes provides a useful framework, but only represents the opposite ends of a wide spectrum of phenotypes which includes an ever-increasing number of subtypes, as revealed by modern single-cell omics technologies. The polarization of macrophages is mediated by a plethora of extrinsic factors such as cytokines, and also by intrinsic regulatory mechanisms, such as increased glycolysis, all of which in various combinations may alter the gene expression profile and reactivity of the macrophages involved [reviewed in (102, 103)]. The metabolic changes in macrophages can be altered by ligand-activated transcription factors such as peroxisome proliferator-activated receptors (PPARs) and liver X receptors. Once activated, these receptors inhibit the pro-inflammatory transcription factors NF-κB and AP-1 and thus downregulate the expression of pro-inflammatory cytokines such as interleukin (IL)-1β, IL-6, and tumor necrosis factor (TNF) (104).

DCs and macrophages phagocytose modified LDL, which the DCs then present to T cells, while the activated macrophages start to secrete cytokines, chemokines, and enzymes, thus further activating the endothelium and leading to increasingly diverse and severe modifications of the trapped lipoproteins (Figure 2).

The modifications of the trapped lipoproteins lead to the formation of DAMPs, which are recognized by pattern recognition receptors (PRRs) located on the surface of antigen-presenting cells, and also on the surfaces of non-immune cells, such as the ECs. The membrane-bound PRRs include toll-like receptors (TLRs), scavenger receptors (72), and the receptor for advanced glycation end-products (RAGE) (105). When activated, the TLRs recruit adapter proteins to activate two main pathways, a MyD88-dependent and a TRIF-dependent pathway, that lead to the production of inflammatory cytokines or type I interferons, respectively (106). Several TLRs have been shown to play important roles in atherogenesis [reviewed in (106)].

Another set of PRRs, the NOD-like receptors (NLRs), are located in the cytoplasmic compartment of cells. Once a cell senses intracellular damage-associated events such as membrane damage, lysosomal rupture, or mitochondrial damage, it initiates the assembly of the NLR family pyrin domain containing 3 (NLRP3) inflammasome (107). Inflammasomes are large cytosolic multiprotein complexes, and once assembled, they induce the activation of inflammatory responses, including proteolytic activation and secretion of the proinflammatory cytokines IL-1β and IL-18 (108). Especially the NLRP3 inflammasome plays a role in atherosclerotic plaque formation [reviewed extensively in (108)]. Inflammasome-mediated activation of caspase-1 can also initiate a form of lytic and highly inflammatory cell death called pyroptosis, in which the protein gasdermin D is cleaved, after which its N-terminus oligomerizes and forms pores in the cell membrane (109). Crystalline cholesterol induces lysosomal damage, and it was the first NLRP3 activator associated with atherosclerosis (57, 75). Crystalline cholesterol can be formed extracellularly from lipoprotein aggregates in the intima, and intracellularly during the lysosomal degradation of modified lipoproteins.

Several studies have shown that modified lipoproteins can activate leukocytes and trigger a pro-inflammatory response (18, 110). This response is leading to the secretion of a plethora of cytokines, chemokines, growth factors, and lipids. The most important ones in the context of this review are shown in Table 1, and the individual responses of cell types to specific modified lipoproteins discussed below are summarized in Table 2.

**Table 1:** Main cellular mediators of atheroinflammation.

**Name**	**Function in atherogenesis**
IL-1β	Drives inflammation during atherogenesis and the evolution of advanced atheroma (108, 111, 112)
IL-1α	Promotes remodeling during early atherogenesis (108, 111)
IL-6	Pro-inflammatory in chronic inflammation, anti-inflammatory in acute inflammation (112–115)
TNF	Pro-inflammatory, activates leukocytes, induces endothelial dysfunction (115, 116)
IL-8	Pro-inflammatory, neutrophil and monocyte chemotactic factor (117, 118)
IL-10	Anti-atherogenic, downregulates production of TNF and ICAM-1 (115, 119)
IL-35	Anti-atherogenic (120)
IL-12	Pro-atherogenic, elevated plasma levels (120)
IL-18	Pro-inflammatory (112)
IFN-γ	Pro-inflammatory, multiple roles at different stages of atherogenesis (121)
MCP-1	Pro-inflammatory, chemotactic activity for monocytes and basophils (122)
CCR2	Chemokine receptor for MCP-1
CXCL1	Pro-inflammatory, chemotactic factor for monocytes (122)
GM-CSF	Induces a pro-inflammatory phenotype in macrophages (123, 124)
TGF-β	Pro-inflammatory (125), but also atheroprotective (126)
PDGF	Intra-plaque angiogenesis, elevated in atherosclerosis (127)
VEGF	Intra-plaque angiogenesis, elevated in atherosclerosis (127)
ICAM-1	Allows rolling and adherence of leukocytes to the endothelium (122)
VCAM-1	Allows rolling and adherence of leukocytes to the endothelium (122)
MMP's	Promote plaque instability by degrading extracellular matrix proteins (112)
p38MAPK	Transcription factor for proinflammatory TNF and IL-1 family signaling (128)
NF-κB	Transcription factor for proinflammatory TNF and IL-1 family signaling (128)
AP-1	Transcription factor for cytokines and growth factors in innate immune response (128)
ROS	Pro-inflammatory, pro-atherogenic or atheroprotective depending on the context (129)
AA	AA metabolites mediate initiation and resolution of inflammation and have been linked to the pathophysiology of many chronic inflammatory diseases (130)
PAI-1	Vascular PAI-1 excess is thought to promote the development of intravascular thrombosis and atherosclerosis (131)
PECAM-1	Adhesion factor, promotes leukocyte extravasation (132)
E-Selectin	Adhesion factor, promotes leukocyte extravasation (132)
P-Selectin	Adhesion factor, promotes leukocyte extravasation (132)

*Interleukin (IL), tumor necrosis factor (TNF), interferon-γ (IFN-γ), monocyte chemoattractant protein-1 (MCP-1), C-C chemokine receptor type 2 (CCR2), C-X-C Motif Chemokine Ligand 1 (CXCL1), granulocyte-macrophage colony-stimulating factor (GM-CSF), transforming growth factor β (TGF-β), platelet-derived growth factor (PDGF), vascular endothelial growth factor (VEGF), intercellular adhesion molecule 1 (ICAM-1), vascular cell adhesion molecule 1 (VCAM-1), matrix metalloproteinases (MMP's), p38 mitogen-activated protein kinase (p38MAPK), nuclear factor kappa-light-chain-enhancer of the activated B-cell (NF-κB), activator protein 1 (AP-1), reactive oxygen species (ROS), arachidonic acid (AA), plasminogen activator inhibitor-1 (PAI-1), platelet endothelial cell adhesion molecule-1 (PECAM-1)*.

**Table 2:** Cellular responses to selected modified lipoproteins.

	**Cell type**	**Response**
vxLDL	Macrophages	IL-1β secretion (133), IL-6 secretion (133, 134), IL-10 secretion (133), TNF secretion (135)
acLDL	Macrophages	IL-1β secretion (133), IL-6 secretion (133, 134), IL-10 secretion (133), C3 secretion (136)
LDL(-)	ECs	Expression of adhesion molecule VCAM-1 (137, 138), ICAM-1 (138), MCP-1 (138, 139), PDGF (140), IL-6 and GM-CSF secretion (140, 141), IL-8 (138–140),
	Macrophages	IL-1β secretion (133), IL-6 secretion (133, 134), TNF secretion (134), IL-8 secretion (134), IL-10 secretion (133)
	Dendritic cells	IL-12 and TNF production (142)
	Monocytes	IL-6 secretion (133, 134), TNF secretion (134), IL-8 secretion (134)
E-LDL	ECs	Expression of IL-8 (143) Expression of IL-8 (93), ICAM-1, PECAM-1, P-selectin, and E-selectin (144)
	Macrophages	Strong synthesis of MCP-1, mild release of IL-6 (145)
	Dendritic cells	TNF secretion (142)
LAL-LDL	Macrophages	IL-8 secretion, activation of transcription factors p38 MAPK and NF-κB (146)
PLA_2_-LDL	ECs	IL-6 and GM-CSF secretion (133, 140, 141), upregulation of E-Selectin, ICAM-1, VCAM-1 (147) and release of arachidonic acid (147)
	Macrophages	IL-1β secretion (56).
SMase-LDL	Macrophages	SMase-LDL treatment increases the lipopolysaccharide-induced secretion of TNF, IL-6, and MCP-1 (84)
oxLDL	ECs	LOX-1 activation and subsequent activation of NF-κB, increase in ROS/decrease in NO, increased expression of MCP-1, VCAM-1, ICAM-1, P-selectin, decreased expression of TGF-β, and apoptosis (148, 149).
	Macrophages	IL-1β secretion (133), IL-6 secretion (133, 134), IL-10 secretion (133), C3 secretion (136)
oxVLDL	ECs	Expression of IL-15, MMP-2, MIF, downregulated expression of TGF-β, ROS production (150), MCP-1 upregulation (151)
	Macrophages	MCP-1 upregulation (151)
	Monocytes	Increased MCP-1 expression (152)
cLDL	ECs	ICAM-1 and VCAM-1 expression (153)
cHDL	ECs	Expression of VCAM-1 and ICAM-1 and increased monocyte adhesion (154).
AGE-LDL	ECs	Expression of ICAM-1,VCAM-1 (155–157), IL-6 secretion (158), TGF-β, TNF, and MCP-1 synthesis (155, 157)
	Macrophages	IL-6 secretion (158)
	Monocytes	Increased expression of MCP-1 receptor CCR2 (159)
	VSMCs	Increased ROS and expression of MCP-1 (160)

*Vortexed LDL (vxLDL), acetylated LDL (acLDL), electronegative LDL (LDL(-)), enzymatically modified LDL (E-LDL), lysosomal acid lipase-modified LDL (LAL-LDL), phospholipase A2-modified LDL (PLA_2_-LDL), sphingomyelinase-modified LDL (SMase-LDL), oxidized LDL (oxLDL), oxidized VLDL (oxVLDL), carbamylated LDL (cLDL), carbamylated HDL (cHDL), advanced glycation end-product-modified LDL (AGE-LDL), endothelial cells (ECs), interleukin (IL), tumor necrosis factor (TNF), complement component 3 (C3), vascular cell adhesion protein 1 (VCAM-1), intercellular adhesion molecule 1 (ICAM-1), monocyte chemoattractant protein-1 (MCP-1), platelet-derived growth factor (PDGF), granulocyte-macrophage colony-stimulating factor (GM-CSF), platelet endothelial cell adhesion molecule (PECAM-1), lectin-type oxidized LDL receptor 1 (LOX-1), nuclear factor kappa-light-chain-enhancer of the activated B-cell (NF-κB), reactive oxygen species (ROS), matrix metalloproteinase (MMP), macrophage migration inhibitory factor (MIF), transforming growth factor β (TGF-β)*.

### Vortexed and Acetylated Lipoproteins

Acetylated LDL (acLDL) and vortexed LDL (vxLDL) are models of modified lipoproteins that have been used to study intracellular lipid accumulation in macrophages, SMCs, and ECs (161–165). Vortexing of LDL leads to aggregation of the particles, but these modifications do not change the lipid moiety of LDL particles and, therefore, experiments in which they have been used provide information merely on their effects on lipid loading on macrophages. Treatment of macrophages with modified LDLs that induce foam cell formation, including acLDL, results in up-regulation of genes involved in the inflammation and immune responses (110).

VxLDL induces mitochondrial dysfunction in macrophages, a known activator of the NLRP3 inflammasome (166). Persson *et al*. studied the basal and inducible cytokine expression in primary human macrophages loaded with cholesterol using vxLDL and they observed that the cholesterol contained in the vxLDL did not affect IL-1β secretion, while the secretion of TNF, IL-6, and IL-8 were significantly decreased (167). However, the amount or composition of neutral lipids in the vxLDL did not affect cellular activation by exogenous TNF, making it likely that lipid loading attenuates cytokine secretion during basal conditions, and that the effects can be overruled by TNF during an acute inflammation (167). Sabeva *et al*., however, reported that IL-1 and IL-6 signaling can be induced by vxLDL in THP-1-derived macrophages (135). Depending on the macrophage polarization, inflammatory responses to cholesterol loading can vary vastly, as recently shown by comparing the inflammatory responses in M1-like and M2-like macrophages upon acLDL loading (89).

### Circulating Modified Lipoproteins

Electronegative LDL (LDL(-)), carbamylated LDL (cLDL) or HDL, glycosylated LDL (AGE-LDL), and mildly oxidized LDL are found in the circulation, and they can initiate foam cell formation in cell culture [reviewed in (168–170)].

LDL(-) is a naturally occurring, minor form of modified LDL in plasma, and it can be isolated based on its differing size, density, and charge. Compared to native LDL, LDL(-) has similar oxidation levels, but it differs in its lipid and protein compositions and in its apoB-100 conformation. LDL(-) also has higher phospholipolytic activities, higher aggregation levels, and higher proteoglycan binding affinity than LDL (133).

In ECs, LDL(-) increases TNF-induced inflammatory responses, such as the expression of transcription factors nuclear factor kappa-light-chain-enhancer of activated B cells (NF-κB) and Activator Protein 1, the expression of Vascular Cell Adhesion Molecule 1 (VCAM-1) (137), and the secretion of the chemoattractants IL-8 and Monocyte Chemotactic Protein 1 (MCP-1) (139), C-X-C Motif Chemokine Ligand 1, the cytokine IL-6, and the growth factors platelet-derived growth factor, Granulocyte-Macrophage Colony-Stimulating Factor (GM-CSF) and Vascular Endothelial Growth Factor (138, 140, 141). In monocytes and macrophages, LDL(-) directly induces priming, inflammasome activation with subsequent IL-1β release (171, 172), as well as secretion of IL-6, IL-8, and TNF (134). Puig *et al*. compared the effects of oxLDL, vxLDL, and acLDL with those of LDL(-) on THP1-derived macrophages and found that while oxLDL induced a more pronounced IL-1β release, LDL(-) induced a stronger release of IL-6, IL-10, and GM-CSF than did other modified LDLs (133). Interestingly, unlike other modified LDL preparations, LDL(-) induced a very strong triglyceride (TG) accumulation in lipid droplets of macrophages (133), a finding which may have clinical significance in that macrophages isolated from human atherosclerotic aorta show significant accumulation of TGs (173).

Protein carbamylation is a post-translational modification of proteins involved in a variety of disease states including inflammation, impaired renal function in chronic kidney disease, and, moreover, elevated levels of carbamylated proteins are present in smokers. Carbamylation has been mechanistically linked to atherosclerosis [reviewed in (174)], especially in patients with chronic renal failure who have vastly higher levels of carbamylated LDL in serum and a higher ASCVD risk (175). It has also been shown to induce endothelial dysfunction (176), increase the expression of adhesion molecules (153), the oxidative stress, and DNA damage in endothelial progenitor cells (177). Also carbamylated HDL increases monocyte adhesion to ECs via inducing NF-κB activation and the expression of VCAM-1 and the intercellular adhesion molecule-1 (ICAM-1) (154). Carbamylation also renders LDL more prone to oxidation, and LDL with both modifications has more pro-atherogenic effects on ECs and macrophages than either modification alone (178).

Non-enzymatic modifications of proteins by glucose are called advanced glycation end-products (AGEs), and they are formed at accelerated rates in hyperglycemic diabetic patients. AGEs can also form on lipids, as evidenced by the observation of lipid-linked AGEs in LDL particles derived from individuals with or without diabetes (179). The AGE-products include circulating AGE-peptides which derive from the catabolism of AGE-modified tissue proteins, and, moreover, AGE-modified LDL is formed by direct reaction between native LDL and circulating reactive AGE-peptides (179). The AGEs, among them also the AGE-LDL, damage vascular cells, one pathway being their interaction with their purported receptors on vascular ECs, SMCs, and macrophages. Among the ensuing implicated diverse pro-atherogenic processes, those leading to endothelial dysfunction have received much attention (155). The adverse endothelial effects include apoptosis (180), reduced nitric oxide synthesis, increased production of reactive oxygen species, and increased expression of (VCAM)-1 (156). Moreover, AGE-LDL particles induce IL-6 secretion by ECs and macrophages (158).

### Phospholipolytically Modified Lipoproteins

The lipolytic products generated by the action of PLA_2_, fatty acids and lysophospholipids, induce proinflammatory responses in cultured human umbilical vein ECs (181). PLA_2_-treated LDL induces exocytosis of the Weibel-Palade Bodies, an early-phase endothelial inflammatory response from ECs in a lysophosphatidylcholine (LPC)-dependent manner (182). LPC acts as a find-me/eat-me signal, which belongs to a group of soluble mediators released by apoptotic cells that attract phagocytes into the tissue (183). PLA_2_-LDL has also been shown to upregulate the adhesion factors E-selectin, ICAM-1, and VCAM-1 in ECs (147). In primed human macrophages, PLA_2_-treated LDL and especially LDL and VLDL treated with both PLA_2_ and LAL have shown to elicit a strong induction of NLRP3-dependent IL-1β secretion (56).

Treatment of LDL particles with SMase, which is secreted by several cell types within the arterial wall, leads to the generation of ceramide and to conformational changes in apoB-100 that induce particle aggregation and increased proteoglycan binding (48–50). These effects could be alleviated by treatment of the LDLs with an apoA-I mimetic peptide prior to the hydrolysis (184). SMase-LDL has been shown to induce the secretion of TNF, IL-1β, IL-6, and the chemoattractant MCP-1 from human macrophages derived from cultured THP-1 monocytes (56, 84). LDL which has been lipolytically modified by secretory PLA_2_ or SMase potentiates the cellular release of proinflammatory lipid mediator arachidonic acid, and triggers activation of the cytoplasmic PLA_2_ in human THP-1 monocytes (185).

Regarding SMase, another point to consider is that it is expressed by macrophages. Endogenous sphingomyelins on the cell membrane can be hydrolyzed, and the generated ceramides can accumulate or be converted into numerous metabolites [reviewed in (186)]. These metabolites are involved in a variety of processes connected to cardiovascular health, they modulate the signaling and metabolic pathways driving insulin resistance, TG production, apoptosis, and fibrosis [reviewed in (187)]. Cellular uptake of SMase-modified LDL brings substantial amounts of ceramides into lysosomes, and, moreover, as LDL contains significant amounts of SM, uptake of modified LDL can also in other ways affect the sphingomyelin pool in macrophages and thus the concentrations of ceramides in them. Kinscherf *et al*. showed in their early work that loading of macrophages with oxLDL and acLDL delivers a substantial amounts of sphingomyelin into the cells and also stimulates ceramide formation in them (188).

### Enzymatically Core-Modified Lipoproteins

Modifications of the lipoprotein core at neutral pH are more difficult to perform due to the protective surface monolayer of the lipoproteins. Therefore, the core-modifying treatments are usually preceded by a modification of the surface. To generate enzymatically modified LDL (E-LDL), LDL is first treated with a protease and subsequently with cholesterol esterase. Such multiply-modified LDL has been detected by immunohistochemical means in human atherosclerotic lesions at all stages of lesion development (55). Treatment of ECs with E-LDL leads to the expression of IL-8 (143), induces the adhesion of monocytes and T lymphocytes to EC monolayers, and stimulates the upregulation of the adhesion factors ICAM-1, platelet-endothelial cell adhesion molecule-1 (PECAM-1), P-selectin, and E-selectin (144). In aortic SMCs, E-LDL upregulates the expression of ICAM-1, which correlates with increased adhesion of T lymphocytes (144). It also strongly induces SMC foam cell formation and upregulates the expression of lectin-like oxLDL receptor 1 (LOX-1) with ensuing increase in the uptake of oxLDL (189).

In macrophages, E-LDL induces strong secretion of MCP-1 and mild release of IL-6 (145). It also promotes monocytic differentiation into dendritic cells, and, when dendritic cells are treated with CRP and/or E-LDL, the production of IL-12 and TNF is significantly increased, and a strong Th1 reaction and T cell proliferation are elicited (142).

LAL hydrolyzes TGs and cholesteryl esters in the core of the lipoprotein particles. LAL is an essential lysosomal enzyme and its deficiency leads to cholesteryl ester storage disease (190). Expression of LAL has been shown to become downregulated in several cell types when the cells are loaded with modified LDL (191, 192). LAL is also secreted from activated macrophages and can then act on lipoproteins also extracellularly (54). Treatment of macrophages with LAL-LDL activates the transcription factors p38 MAPK and NF-κB, induces the secretion of IL-8 (146), and may also promote vascular dysfunction and atherogenesis [reviewed in (193)]. LDL and VLDL, when treated first with enzymes that hydrolyze the particle surface before treatment with LAL are able to trigger a robust, NLRP3-mediated IL-1β secretion in human macrophages (56).

VLDL remnants treated with lipoprotein lipase to hydrolyze the TGs have been shown to induce increased expression of TNF, IL-1β, and IL-8 over native VLDL or lipoprotein lipase, with concurrent activation of NF-κB and activator protein 1 in peripheral blood mononuclear cells and THP-1 monocytes (194).

### Oxidized Lipoproteins

Oxidation is the most studied form of lipoprotein modification, and the pro-inflammatory effects of oxidized phospholipids in inflammation and various inflammatory diseases are well established (195).

OxLDL acts as a DAMP and triggers sterile inflammatory responses (195). In ECs it causes endothelial dysfunction and induces a pro-inflammatory and pro-thrombotic state (196). It also enhances monocyte chemotaxis and adhesion to EC in culture (197). The uptake of oxLDL by macrophages is mediated by scavenger receptors such as CD36, and it induces the assembly of a TLR4/TLR6 heterodimer, and results in NF-κB signaling and priming of these cells for inflammasome activation (198). OxLDL uptake by CD36 additionally results in intracellular cholesterol crystallization and can thereby directly activate the NLRP3 inflammasome in macrophages (88). Both minimally oxidized LDL and more highly oxidized LDL cause the secretion of pro-inflammatory cytokines by macrophages (70, 71) by activating TLR4 (199, 200). Li *et al*. showed in THP-1 monocytes that oxLDL and oxHDL increases the expression of NLRP3 and activates caspase-1 and induces IL-1β and IL-18 secretion in a dose-dependent manner (201). However, other studies have shown that oxLDL inhibits the production of inflammatory cytokines by macrophages in response to inflammatory stimuli, such as LPS (202). More recently, it was shown that oxLDL, as well as oxHDL3, reduces the secretion of mature IL-1β by inhibiting the activation of the NLRP3 inflammasome induced by known inflammasome activators including the acute-phase serum amyloid A protein, ATP, nigericin, and monosodium urate crystals (203). By binding to LOX-1 on SMCs, oxLDL can also induce SMC transition to an inflammatory phenotype. Thus, treating cultured SMCs with oxLDL stimulates the expression of the chemokines MCP-1 and C-X-C Motif Chemokine Ligand 1, the cytokine TNF, and the cell adhesion molecule VCAM-1 [reviewed in (204)].

OxLDL can also induce pyroptosis in macrophages (205) and ECs (206), and oxLDL-induced pyroptosis has also been implicated in endothelial dysfunction and vascular SMC foam cell formation [reviewed in (109)].

OxLDL is also able to induce a novel form of cell death coined ferroptosis, which is linked to atherogenesis (207, 208). Bai *et al*. showed that some of the key morphological features of ferroptosis, such as mitochondrial shrinkage, increased iron content, increased release of lactate dehydrogenase, and reduced expression of the solute carrier family 7 member 11 and glutathione peroxidase 4 were induced by oxLDL in mouse aortic ECs to a comparable amount achieved with the ferroptosis inducer erastin; the phenotype was rescued by co-stimulation with ferroptosis inhibitor ferrostatin-1 (209). Further, oxLDL- and erastin-treated cells had similarly increased levels of markers of intracellular lipid peroxidation, such as total ROS, lipid ROS, lipid peroxide, and malondialdehyde, while treatment with ferrostatin-1 downregulated the generation of these lipid peroxidation products (209). Similar induction of ferroptosis by oxLDL was observed in human coronary artery ECs (210).

While minimally oxidized LDL is found in circulation, the vast majority of circulating oxLDL is bound to antibodies and found as oxLDL immune complexes, which can prime dendritic cells and macrophages for inflammasome activation (211). While the circulating immune complexes may be too large to cross an intact endothelium, immune complexes can also be formed within the arterial intima (212). Interestingly, immune complexes composed of oxLDL and anti-LDL IgG antibodies can trigger activation of cultured human mast cells, an innate immune type of cell present in human atherosclerotic lesions, and, when activated, capable of proteolytically modifying LDL and HDL particles (213, 214).

In ECs, oxidized VLDL initiates the expression of the fibrinolysis inhibitor plasminogen activator inhibitor-1 and stress response protein heat shock factor 1 compared to the effects obtained with VLDL or LDL (215), and induces endothelial apoptosis (216). It also induces expression of IL-15, matrix metalloproteinase-2, macrophage migration inhibitory factor, as well as downregulated expression of transforming growth factor beta (TGF-β) (150). In macrophages, both oxidized VLDL and its remnants induce foam cell formation that can be attenuated by pre-incubation with TGF-β (217). In both monocytes and macrophages, oxidized VLDL induces the expression of MCP-1 (151, 152).

Oxidized HDL (oxHDL) plasma levels have been shown to correlate with ASCVD and coronary artery calcification [recently reviewed in (218)]. OxHDL induces endothelial dysfunction in ECs by a positive feedback mechanism in which an oxidative burst generates oxHDL from native HDL, activates LOX-1 which in turn increases the expression of NADPH oxidase 2, TNF, and LOX-1 receptor at the endothelial plasma membrane (219). It has also been shown to decrease the expression of CD63 in macrophages, thus attenuating the ability of the cell to ingest modified lipoproteins (220, 221). OxHDL also binds to CD36 on platelets and induces proinflammatory and procoagulant effects (222).

### Neutrophil Extracellular Traps

Another innate immune pathway contributing to vascular inflammation is neutrophil extracellular trap (NET) formation, also called NETosis (223). NETs are structures of chromatin filaments coated with histones, proteases, as well as granular and cytosolic proteins, which are ejected by activated neutrophils to immobilize and eliminate pathogens as a part of the first-line immune defense.

Recent research has revealed that NETosis is associated with the initiation and progression of various noninfectious diseases (224), and NETosis has also been shown to promote thrombosis (225). NETs have been found in vascular lesions such as atherosclerotic plaques (226), and infiltration of neutrophils into arteries during the early stages of atherosclerosis has been observed in hypercholesterolemic mice (227). One theory about the reason for NETs in plaques is that periodontal pathogens enter the bloodstream from severely infected periodontal tissues, then reach the inflamed atherosclerotic lesions where they can directly contribute to the development of the lesions [reviewed in (228)].

Non-infectious triggers such as LDL-derived cholesterol crystals might also induce NETosis in atherosclerosis. NETs prime macrophages, which in turn start to produce pro-IL-1β and IL-8 (117). Cholesterol crystals can bind to CD36 on the macrophages and thereby trigger inflammasome activation with the ensuing secretion of mature IL-1β. Thus, NETs may contribute to atherogenesis by initially activating macrophages (229, 230). Studies in apoE-/- mice with deletions of two neutrophil proteases showed attenuated NETosis as well as smaller atherosclerotic lesions, and lower systemic IL-1β levels, when compared to ApoE–/– mice expressing these proteases (230), indicating a potential role for NETs and/or these proteases in this process.

Kato *et al*. demonstrated the appearance of oxLDL in vascular tissues and circulation even prior to atherosclerotic lesion development in apoE-KO mice (231). OxLDL as well as its various oxidized phospholipid components, especially oxPCs and LPC, mediate NETs formation and subsequent endothelial inflammatory responses in both HL-60-derived neutrophils and human polymorphonuclear neutrophils (232, 233).

## Adaptive Immune System Response and Autoimmune Component

Adaptive immunity is heavily involved in atherogenesis [reviewed in (234)] and comprises a humoral component of specific antibodies produced by B cell-derived plasma cells, and a cellular component with T cells that either activate B cells or differentiate into effector T cells, which are matured by being presented antigens by antigen-presenting cells. Of the T cells, those expressing CD8 mature into cytotoxic CD8+ T cells and those expressing CD4 into helper CD4+ T cells or regulatory T cells. Both CD4+ and CD8+ T cells are recruited into atherosclerotic lesions and display signs of activation (235). On the other hand, the regulatory T cells comprise the only T cell subset that has only negative regulatory effects on the autoimmune response, and therefore also a dampening influence on atheroinflammation. The recruitment of regulatory T cells into the arterial intima is promoted by the activation of the nuclear receptor peroxisome proliferator-activated receptor γ (PPAR-γ), which can further exert anti-atherosclerotic effects by inhibiting the expression of various pro-inflammatory factors, improving the function of endothelial cells, inhibiting MCP-1 expression, and restraining the differentiation of monocytes into macrophages [reviewed in (236)].

T cells with a specificity for apoB-derived epitopes have been identified, so linking adaptive immune responses to the vascular retention of LDL (237). Vaccination of humanized atherosclerotic mice with two epitopes from human native apoB-100 that trigger T-cell activation protected the mice against atherosclerosis (238). T cells constitute about 10% of all cells in human plaques, and 70% of them have been described to be CD4+, and the remaining largely CD8+ T cells (239).

The final stage of B cell maturation into antibody-producing plasma cells occurs after their activation. There are two main subtypes, B1 cells which produce the majority of natural IgM and to a lesser extent IgG, and B2 cells which can differentiate directly into memory B cells (240). T cells and macrophages are found in all stages of atherosclerosis, whereas B cells are only occasionally found in the atherosclerotic plaques themselves. Rather, a larger number of B cells may be found in the adventitial layer of the arterial wall, where tertiary lymphoid organs can be formed (241). Resident B2-type lymphocytes isolated from human carotid atherosclerotic walls have been found to be mainly activated plasmablasts lacking terminal differentiation to plasma cells, and preferentially expressing IgG and IgA (242).

Functionally, the effects of B and T cells in atherosclerosis, the primary site of lipoprotein modification, are mixed. In a recent consensus paper (6) the involvement of lymphocytes is described as follows: Dendritic cells take up modified LDL and present specific epitopes such as apoB peptides to naive T cells, inducing their differentiation into CD4+ helper T cells (243). These CD4+ T cells, together with the specific cytokines that they secrete, provide help to B cells, and regulate the activity of other T cell subtypes. The pro-atherogenic role of interferon gamma (IFN-γ)-secreting Th1 cells and the anti-atherogenic effect of IL-10/TGF-β-secreting T regulatory cells are well established (244). Cytotoxic CD8+ T cells have been shown to promote atherogenesis (245). Hermansson *et al*. studied the mechanism of T cell recognition of LDL particles by generating T cell hybridomas from human apoB-100 transgenic mice that were immunized with human oxLDL, and found that none of the hybridomas responded to oxLDL, but, instead, to native LDL and isolated apoB-100. However, sera from these immunized mice contained antibodies against oxLDL, suggesting that T cell responses to apoB-100 aid B cells to generate antibodies against the epitopes present in both native LDL and oxLDL. ApoB-100-responding CD4+ T cell hybridomas expressed a single T cell receptor variable β chain, and immunization of humanized atherosclerotic mice with a peptide derived from that T cell receptor-induced antibodies that blocked T cell recognition of apoB-100, significantly reduced atherosclerosis with a concomitant reduction of macrophage infiltration in the lesions (246). Ruuth *et al*. observed similar inhibition of T cell activation by oxLDL, but they found that SMase-LDL activated T cells and that the degree of LDL aggregation induced by SMase associated positively with the degree of T cell activation, as measured by the secretion of interleukin-2 (22). Up to 10% of CD4+ T cells isolated from human atherosclerotic plaques displayed specificity for oxLDL (247).

If incubated with whole blood, native and vxLDL elicit vastly different T cell activation, native LDL activating CD4+ cells only to a small extent, and vxLDL potently activating CD8+ cells (248). The response might be indicative of *in vivo* formed intimal aggregation of modified LDL.

Distinct roles for different B cell subsets have been reported, anti-oxLDL immunoglobulin IgM antibodies produced by B1 cells being atheroprotective, and anti-oxLDL IgG antibodies produced by B2-cell subsets likely being pro-atherogenic (237, 249, 250). Although only small numbers of B cells are found in atherosclerotic lesions, both IgG and IgM antibodies derived from such cells accumulate in the lesions. Recently, Upadhye *et al*. identified bone marrow B-1a cells that contribute abundantly to IgM production with a unique IgM pool, which includes anti- malondialdehyde-modified LDL (MDA-LDL) antibodies. They also showed that expression of the chemokine receptor CXCR4 is a critical factor for the B-1a localization and production of IgM against oxidation-specific epitopes. Expression of CXCR4 on human B-1 cells was greater in humans with low coronary artery plaque burden, suggesting an antiatherogenic function for the cells (251).

### Natural IgM vs. IgG

Natural antibodies are antibodies that spontaneously arise without prior infection or defined immune exposure. Most serum antibodies at birth are IgM natural antibodies and, as such, they represent a primitive innate-like “layer” of the adaptive immune system (252, 253). Antigens that are specifically recognized in atherosclerosis include microbial antigens, endogenous heat shock proteins, β-2 glycoprotein I, and modified LDL, especially oxLDL (253). The degree of oxidation of LDL in humans is variable, and two major modifications prepared *in vitro*—copper-oxidized LDL and MDA-LDL—are recognized by human autoantibodies. Both forms share MDA-lysine as their main oxidation-specific epitope but MDA-LDL contains roughly a 10-fold higher amount of the epitope (254).

IgG and IgM antibodies against oxidation-specific epitopes of oxLDL such as MDA-modified lysine residues or the phosphorylcholine headgroups of oxidized phospholipids have also been found in the plasma, and oxLDL-immune complexes are present in plasma (255) as well as in atherosclerotic lesions of humans and animals (212). Several clinical studies investigated the association of oxLDL autoantibodies with atherosclerosis progression, and in most, atherosclerosis or its progression was directly associated with oxLDL IgG levels, and inversely associated with IgM (256, 257) [reviewed in (240)]. While oxidation-specific epitopes are also commonly found on surfaces of apoptotic cells, microvesicles, and bacteria (195), antibodies against the epitopes were shown to have the capacity to block oxLDL uptake (258), the case for their role in atherogenesis being strong.

Antibodies against carbamylated LDL have also been found in individuals with uremia or tobacco smoking, and these antibodies also cross-react with MDA- and malondialdehyde acetaldehyde-LDL (259, 260). Further, also IgG against AGE-LDL has been found in human serum and to be associated with diabetes mellitus (261).

## Lipid Mediators in Modified Lipoproteins Impair Resolution

Inflammation is usually followed by its resolution, an active process that reduces leukocyte recruitment, stimulates efferocytosis, repairs tissue damage, and ultimately restores tissue homeostasis. It is partly mediated by proteins such as the anti-inflammatory cytokine IL-10 (119), endogenous gases such as nitric oxide (262), and also by regulatory T cells (263). In recent years, specialized pro-resolving lipid mediators, among others, were discovered to play major roles in mediating the resolution of atherosclerotic lesions [reviewed in (264)]. In the progressing atherosclerotic lesions, efferocytosis of lipid-laden apoptotic cells is impaired, a malfunction that leads to chronic inflammation and a constant influx of circulating monocytes, thus promoting the growth of a necrotic lipid core (263). In macrophage foam cells of atherosclerotic plaques, the lipid mediators, such as 5-lipoxygenase, show nuclear localization, thereby shifting their metabolism to promote the generation of pro-inflammatory leukotrienes and to decrease the production of specialized pro-resolving mediators (265).

OxLDL has been shown to impair efferocytosis in mouse macrophages and to induce apoptosis (266), potentially due to oxidized phospholipids [reviewed in (267)] and cholesterol (268) which are potent mediators of inflammation and influence the ability of the macrophages to perform this function of cell removal. Copper-oxidized LDL was also found to contain lipofuscin-like fluorophores (269), known inducers of the NLRP3-inflammasome (270) and of necroptosis (271). Oleic and linoleic acids associated with E-LDL induced IL-8 expression in ECs (143). Ceramides arising from SMase activity have been shown to inhibit efferocytosis by alveolar macrophages (272, 273). PLA_2_-LDL induces the release of arachidonic acid from ECs (147). Arachidonic acid can be converted into prostaglandins, thromboxanes, and leukotrienes, all of which are potent modulators of inflammation. PLA_2_ treatment also releases LPC which is a well-known chemotactic eat-me signal during apoptosis, and which also increases the expression of ICAM-1 and VCAM-1 in ECs and enhances monocyte binding to the endothelium (274). Thus, modified lipoproteins and their cargo directly contribute to impaired resolution in atherogenesis.

## Dysfunctional HDL

HDLs are considered antiatherogenic [reviewed in (275)]. Firstly, low levels of HDL-C are present in many conditions that are associated with an increased ASCVD risk. The most prominent antiatherogenic property associated with HDL is their ability to induce macrophage cholesterol efflux. HDL also enhances the endothelial synthesis of the vasodilator nitric oxide and thus can also ameliorate endothelial dysfunction. Furthermore, HDL reduces coronary atherosclerosis by decreasing the expression of adhesion molecules on ECs and thereby reducing the infiltration of pro-inflammatory cells and LDL into the subendothelial space. HDL also inhibits lipid oxidation and exerts anti-inflammatory and antiapoptotic actions [reviewed in (275)].

However, oxidative stress can modify HDL. Within the subendothelial space, apoA-I can be oxidized on multiple residues by the proinflammatory enzyme myeloperoxidase (275, 276), and the resulting dysfunctional HDL is associated with an increased incidence of cardiovascular events [reviewed in (218)]. During inflammatory states, inflammatory cytokines induce hepatic expression of the acute-phase serum amyloid A (277) and group IIa sPLA_2_ (278), which then leads to the formation of HDL particles that are relatively enriched in SAA, while depleted of apoA-I and phospholipids. Also, increased oxidative stress during inflammation generates dysfunctional HDL that contains oxidatively modified or carbamylated apoA-I.

HDL remodeling during inflammation also leads to the loss of HDL-associated enzymes such as serum paraoxonase and arylesterases 1 and 3 that normally impede peroxidation of LDL particles; so, the loss of these enzymes decreases the antioxidative and anti-inflammatory capacity of HDL (279).

ApoE-/- mice with dysfunctional HDL displayed an increased cholesterol efflux capacity and lipoprotein peroxidation when treated with an apoA-I mimetic peptide (280). This peptide has also been able to attenuate LDL aggregation after hydrolysis by SMase provided the peptide was added prior to hydrolysis (184).

Impaired cholesterol efflux capacity of HDL correlates with the severity of atherosclerosis, including the early stages of disease development (281, 282). Thus, cholesterol efflux and resulting foam cell formation can be attributed to the dysfunctional HDL rather than the alterations of circulating levels of normal HDL particles, making dysfunctional HDL a better biomarker of atherosclerosis than the level of HDL-cholesterol (168). Mast cells are present in atherosclerotic lesions where they become activated to secrete heparin-bound neutral proteases capable of degrading apoA-I (283). The mast cell proteases avidly degrade lipid-poor HDL particles and thereby prevent their ability to induce cholesterol efflux from macrophage foam cells, i.e., the first step of reverse cholesterol transport (283). We consider that the HDL-modifying enzymes and agents present in inflamed atherosclerotic lesions are the most critical components of the malfunctioning reverse cholesterol transport system. Accordingly, emerging knowledge and understanding of the various steps of reverse cholesterol transport, particularly of the initiation step when macrophage foam cells and HDL particles interact in an atherosclerotic lesion, is crucially important when attempting to develop novel HDL-targeted therapies (284).

## Clinical Implications of Inflammation Induced by Modified Lipoproteins

As discussed in this Review, the apoB-containing lipoprotein particles and the lipids contained in them are critical players in atherogenesis. A large and overwhelming body of evidence strengthens the causal role for the cholesterol contained in the LDL particles throughout all stages of atherogenesis (285). Indeed, without cholesterol there is no atherosclerosis. Of particular clinical significance are the clinical observations on lifelong high or low concentrations of LDL-cholesterol (LDL-C). Thus, in patients with familial hypercholesterolemia, the genetic absence of LDL-receptors, which normally remove LDL particles from the circulation, leads to high LDL-C concentrations since birth and inevitably results in accelerated development of coronary atherosclerosis and its clinical sequelae, such as acute myocardial infarction (286). In contrast, individuals with a life-long history of low LDL-C levels due to genetic defects in the function of the LDL-receptor-suppressing PCSK9 molecule have a dramatic reduction in coronary heart disease even in the presence of other risk factors (287). Finally, the ability of therapeutic lowering of LDL-C has proven the essential role of cholesterol in atherosclerosis and its clinical sequelae (288).

Considering the multitude of inflammatory pathways involved in atherogenesis as discussed here, we wish to add that there is no atherosclerosis without superimposed inflammation in the arterial wall. The contribution of inflammation to the development of atherosclerosis has received support from the findings that statin drugs exert anti-inflammatory effects on atherosclerotic lesions and reduce clinical events due to coronary atherosclerosis, and that part of these beneficial effects may be independent of LDL-C lowering (289). The clinical trials have also demonstrated that many patients remain at increased risk because of persistent elevation in high-sensitivity CRP despite significant reductions in LDL-C level, and that this “residual inflammatory risk” is a viable pharmacologic target of ASCVD (290).

Recent trials have successfully demonstrated the ability of anti-inflammatory interventions to reduce recurrent cardiovascular events even in patients with optimal control of LDL-C level (291). In the Canakinumab Anti-inflammatory Thrombosis Outcomes Study (CANTOS) (292), the pro-inflammatory cytokine IL-1β was inhibited by administering systemically the monoclonal antibody canakinumab to patients after myocardial infarction, and a reduction of recurrent major adverse cardiovascular events was observed (292). Since then, clinical trials testing anti-inflammatory compounds have shown beneficial effects on cardiovascular outcomes. Reduction of cardiovascular events has been observed in several trials involving, in addition to the state-of-the-art lipid-lowering drug regimen, the broad anti-inflammatory drug colchicine. Thus, colchicine was used in the Colchicine Cardiovascular Outcomes Trial (COLCOT) in individuals with a recent myocardial infarction (293), and in the low-dose colchicine trials LoDoCo1 (294) and LoDoCo2 involving individuals with clinically chronic stable coronary disease (295). The pro-inflammatory cytokine IL-6 was targeted by the specific antibody tocilizumab in the ASSessing the effect of Anti-IL-6 treatment in MI (ASSAIL-MI) trial (296, 297), and the treatment attenuated the inflammatory response and the degree of myocardial damage in patients with non-ST-elevation myocardial infarction. Moreover, after acute myocardial infarction hydroxychlorine reduced the levels of IL-6 at least for a period of 32 months suggesting that this anti-inflammatory drug may reduce cardiovascular events after such an event (298). However, other anti-inflammatory drugs, like the immunosuppressant methotrexate (299) or the p38 mitogen-activated protein kinase (MAPK) inhibitor Losmapimod (300) did not show any effects revealing the specificity of the clinically relevant anti-inflammatory pathways. Of concern are the observations that systemic anti-inflammatory treatments with IL-1β antibodies increased the individual's susceptibility to fatal infections (301), and that the COLCOT colchicine trial saw a small increase in pneumonia (291).

Interestingly, the adverse effects of air pollutants may occur to a large part via generation of ROS and reactive nitrogen species from organic chemicals present in the inhaled particulate matter (302). Pulmonary oxidative stress and inflammation also results from the activation of pulmonary epithelial cells, macrophages, neutrophils, and ECs by the particulate matter deposited in the lungs. Moreover, the ultrafine particulate matter can reach the systemic circulation where the particles have the potential to directly oxidize HDL or other circulating lipoproteins. Taken together, the pollutants promote the generation of oxLDL, oxHDL, and induce a prothrombotic dysfunction of the coronary endothelium. By causing systemic oxidative stress and inflammation, the air pollutants form one arm in the “common soil” of the cardio-pulmonary continuum (303).

Regarding the development of diabetic vascular injury, the cellular responses to AGEs are thought to largely depend on the interaction between the AGE and AGE-specific cell surface receptors, the RAGEs, which *via* activation of the transcription factor NF-κB leads to a range of proinflammatory reactions and induction of oxidant stress in the cells involved (304). Since AGE formation may lead to oxidative stress and oxidative stress may accelerate the formation of specific AGEs, both AGE-LDL and ox-LDL could be involved in the accelerated development of atherosclerosis in patients with type 2 diabetes (305, 306). Based on the above findings, we may postulate that the observations of specific AGEs being associated with the severity of subclinical atherosclerosis and macrovascular complications in patients with long-standing type 2 diabetes, and the requirement of long-standing glucose-lowering (for 10 years) to lower cardiovascular risk, both could at least partly depend on the generation of AGE-LDL in these patients (307, 308). Indeed, among the molecular mechanisms of how hyperglycemia promotes atherosclerosis, the direct protein-modifying effects of glucose appear to be prominent, the glycated LDL particles being both pro-inflammatory and foam cell-forming species of modified lipoproteins (309).

The atherogenic potential of cholesterol in TG-rich lipoprotein particles (TGRLs) and their remnants is being increasingly acknowledged (310–313). Indeed, elevated remnant cholesterol associates with increased risk for acute myocardial infarction, ischemic stroke and, even stronger for peripheral arterial disease, which is also considered to have a strong atherosclerotic component in its pathogenesis (314). The clinical significance of the TGRL remnants has been mostly assigned to their ability to enter the arterial intima and to contribute to the formation of foam cells. However, the mechanisms by which postprandial lipemia induces atherosclerosis also involve induction of endothelial dysfunction, oxidative stress, and inflammation (315). Importantly, the lipoprotein lipase -mediated hydrolytic modification of the TGRLs takes place not only in capillaries but also in atherosclerosis-susceptible arterial segments where it results in high local concentrations of lipolytic products, such as oxidized free fatty acids which jointly with the TGRLs cause a multitude of inflammatory reactions in the ECs and the cells within the intima (316, 317).

## Concluding Remarks and Future Directions

**Figure 3:**
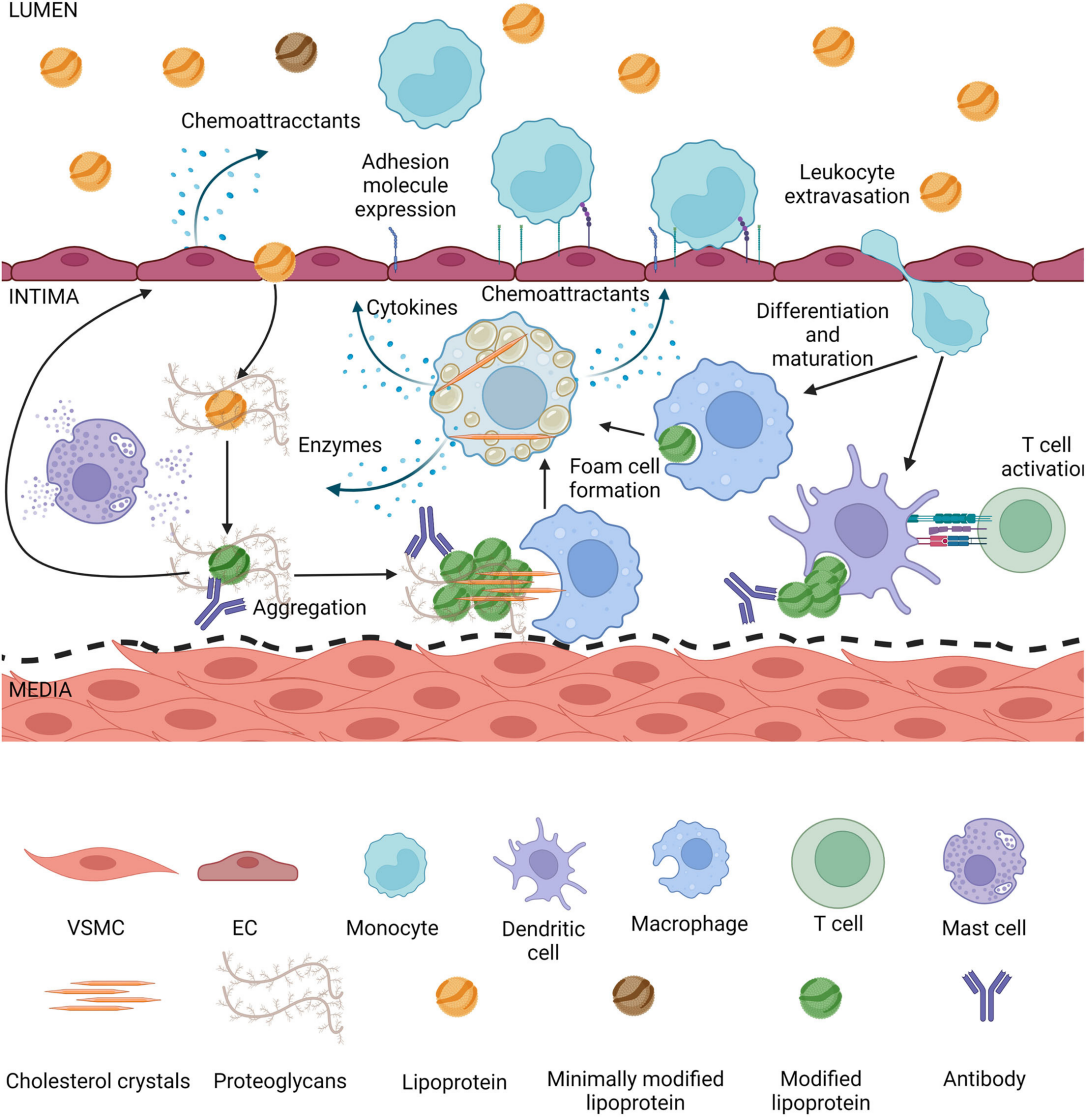
Lipoproteins modified in the intima induce inflammation. Lipoproteins smaller than 80 nm can enter the intima by transcytosis, they get trapped by proteoglycans and during retention they are exposed to enzymes secreted by intimal cells. Modified lipoproteins tend to aggregate, depending on the modification cholesterol crystals can form. Modified lipoproteins also are recognized by antibodies and form immunocomplexes in the intima. The aggregates and crystals can be phagocytized by macrophages or dendritic cells, which can form foam cells or activate T cells, respectively. Mast cells are degranulated during atherogenesis, leading to matrix degradation and lipoprotein degradation. Activated macrophage foam cells secrete more enzymes thus increasing the rate of modification, they secrete cytokines inducing an inflammatory state in the intima, as well as chemoattractants. Endothelial cells either directly by contact with modified lipoproteins or due to pro-inflammatory cytokines from foam cells themselves secrete chemoattractants and increase expression of adhesion molecules, increasing the rate of leukocyte extravasation into the intima. Endothelial activation and dysfunction also lead to a higher rate of lipoproteins entering the intima.

Modified lipoproteins are the main drivers of atherogenic inflammation of the arterial wall (Figure 3). To decrease the vascular wall inflammation due to modified lipoprotein several concepts are being discussed. Without the entry of cholesterol-containing apoB-lipoproteins into the arterial wall, there is no atherosclerosis. Moreover, it is the current consensus that local inflammation in the evolving atherosclerotic lesions is an inherent part of atherogenesis. Regarding the therapeutic options of atherosclerotic cardiovascular diseases, the lipid-lowering and anti-inflammatory therapies do not compete, but they are complementary and compatible. Currently, a myriad of new treatment options of both dyslipidemia and inflammation in ASCVD is emerging, to mention the availability of RNA-based inhibitors of the hepatic synthesis of apoB-containing lipoproteins which could be administered even once a year (318). Moreover, ASCVD-related events could be substantially reduced by prescription-grade eicosapentaenoic acid in patients with hypertriglyceridemia (319). In that study, the beneficial effect likely at least to some extent resulted from lowering cholesterol accumulation and reduction of inflammation in the arterial wall *via* changes in composition of lipoproteins and cells.

Finally, the perception that inflammation would replace traditional risk factors of atherosclerosis poses a false dichotomy (320). Currently, lowering the concentration of the apoB-containing lipoproteins and their remnants either by dietary or pharmacological means is the mainstream approach to combat atherosclerosis both in the primary and secondary prevention settings, while the anti-inflammatory therapies serve as adjunct therapies in the setting of secondary prevention strategies. This chronology adopts our traditional understanding of cholesterol accumulation being the primary event and an inflammatory reaction being a secondary event taking place in the arterial intima, the concept originating in the early work dealing with animal models of atherosclerosis. This basic concept is also reflected in the present-day clinical approach in which the inflammation is considered to present a residual cardiovascular risk after very efficient lowering of LDL-C in patients with clinically manifest ASCVD (321). However, as discussed in this review in-depth, we may consider the cholesterol- and TG-containing lipoproteins entering the arterial intima as innocent bystanders, who are attacked by the “healthy” resident macrophages which have been present in the intima lifelong, and which are performing their physiological patrol tasks in this tissue like they do in any tissue (322). Then, it is, after all the close encounter of the two which ignites and sustains the atherogenic process in which cholesterol accumulation and inflammation coexist. This novel understanding is necessary when aiming at developing new anti-atherosclerotic therapies aiming at primary prevention of ASCVD, as it dictates that the inflammatory component is not only a residual risk but a risk existing throughout the development of atherosclerosis. Recent results obtained in mice with early atherosclerosis revealed that inhibition of IL-1β and NLRP3 inflammasome reduces leukocyte accumulation in atherosclerotic aortas (323). Thus, we may predict that cardiovascular interventions utilizing lowering the levels of circulating apoB-containing lipoproteins, prevention of their modifications, and reduction of the numbers of lesional macrophages already at an initial or early stage of the long subclinical asymptomatic phase of atherosclerosis may ultimately provide us the tools required for full eradication of the clinically manifest ASCVD.

## Author Contributions

ML, KÖ, and PK designed, wrote, and edited the manuscript. All authors contributed to the article and approved the submitted version.

## Funding

Wihuri Research Institute was maintained by the Jenny and Antti Wihuri Foundation. This study was also supported by grants from the Academy of Finland (#332564), Novo Nordisk Fonden (#NNF19OC0057411), the Finnish Foundation for Cardiovascular Research, and the Aarne Koskelo Foundation.

## Conflict of Interest

The authors declare that the research was conducted in the absence of any commercial or financial relationships that could be construed as a potential conflict of interest.

## Publisher's Note

All claims expressed in this article are solely those of the authors and do not necessarily represent those of their affiliated organizations, or those of the publisher, the editors and the reviewers. Any product that may be evaluated in this article, or claim that may be made by its manufacturer, is not guaranteed or endorsed by the publisher.
